# Uptake and Transport of Different Concentrations of PPCPs by Vegetables

**DOI:** 10.3390/ijerph192315840

**Published:** 2022-11-28

**Authors:** Yongfu Zeng, Yiming Zhang, Haichao Zhang, Jing Wang, Kaoqi Lian, Lianfeng Ai

**Affiliations:** 1 School of Public Health, Hebei Medical University, Shijiazhuang 050017, China; 2 Wellington Livestock Pty. Ltd., Cobains, VIC 3851, Australia; 3 Shijiazhuang Customs Technology Center, Shijiazhuang 050051, China

**Keywords:** PPCPs, contaminants, sprouts, plants

## Abstract

In many parts of the world, water resources are scarce or even extremely scarce, and the reuse of water resources has become mainstream in today’s world. Many regions use treated wastewater for agricultural irrigation, aquaculture, and other activities. However, in recent years, wastewater has been found to contain large amounts of pharmaceuticals and personal care products (PPCPs). Therefore, there is a potential risk of PPCPs being transported in the environment and affecting human health. In this study, we compared the uptake, transport, and accumulation of 27 PPCPs in three types of sprouts (radish, buckwheat, and okra).The bioaccumulation of amantadine, diphenhydramine, chlorpheniramine maleate, sibutramine, hemosibutramine, chlorosibutramine, N-monomethyl sibutramine, N, N-desmethyl sibutramine, and carbamazepine was found to be significantly higher in plants grown for 12 days in media containing 0.5, 5.0, and 50.0 ng/mL PPCPs. With increasing concentration of PPCPs in the culture solution, the amount of PPCPs absorbed by plants and the degree of accumulation also showed an increasing trend. At the same time, it was demonstrated that there was an obvious uptake transfer phenomenon of PPCPs by plants, and the trend of uptake transfer became more and more obvious as the concentration of external environmental pollutants increased. In addition, amantadine, chlorpheniramine maleate, carbamazepine, N, N-desmethyl sibutramine, hemosibutramine, and chlorosibutramine showed more active translocation in some plants (TF > 1.0).

## 1. Introduction

Pharmaceutical and personal care products (PPCPs), an emerging contaminant, includes medical drugs, cosmetic ingredients, pesticides, veterinary drugs, and food additives [1]. It originates from expired medications and daily care products in the sewage system, treated and untreated hospital and veterinary hospital waste discharged to the domestic sewage system, aquaculture medicated feed, and releases from industrial waste streams [2]. Not all PPCPs are newly emerging compounds; some have been present in wastewater for decades and have not gained attention until now. In 2004, Daughton suggested that there are approximately six million commercially available PPCPs substances worldwide and that drug use is growing 3.0–4.0% per year [3]. According to statistical reports, there are more than 8000 kinds of preparations in the European market for hygiene products alone. In recent years, as urbanization accelerates, the demand for personal care and hygiene is on the rise, and the use of various drugs, care products, and hygiene products is increasing. Therefore, the risk of environmental pollution by PPCPs may increase.

**Figure 1:**
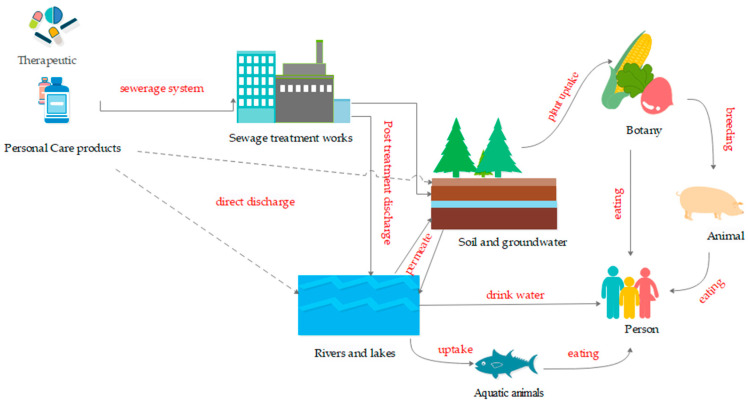
Sources of PPCPs in the environment.

The pollution of the environment by PPCPs is mainly caused by human activities, and the general route of their entry into the environment is shown in Figure 1. Chemical substances such as expired drugs, personal care products, additives, and metabolites from households, hospitals, farms, and factories are discharged into rivers, lakes, groundwater, and soil after treatment in municipal drainage systems, or are discharged directly into the environment without treatment, where PPCPs are absorbed and accumulated by plants and aquatic animals. It has been demonstrated in the literature that wastewater treated in wastewater treatment plants still contains a variety of PPCPs [4,5,6]. Studies on groundwater have shown that drugs such as carbamazepine and primidone are still present in the soil after 8–10 years, and it has also been confirmed that PPCPs bioaccumulate in fish farming downstream of sewage treatment plants [7]. PPCPs were also reported in macroalgae, barnacles, and fish from contaminated coastal waters of the Saudi Red Sea in a related study [8]. A study by Yiyi Yu et al. reported that compounds such as the antiepileptic drug carbamazepine and its metabolites, the X-ray contrast agent iopromide, synthetic hormones, and hormone-based personal care products were detected in sewage, sludge, and urban rivers in the Pearl River Delta of South China, and concluded that wastewater treatment plants are a major source of PPCPs in the environment [9]. A large number of PPCPs were also found in a study of water samples from Saudi Arabia’s Red Sea coastal waters, with metformin, diclofenac, acetaminophen, and caffeine being the most abundant [10]. Although PPCPs are present at ng/L-µg/L concentrations, numerous studies have shown that PPCPs can adversely affect non-target aquatic organisms [11]. Therefore, PPCPs have been of increasing environmental concern in recent decades. 

PPCPs have become a key public concern due to their widespread environmental contamination [12] and potential adverse effects on human health and organisms in the environment [13]. Related studies have shown that once PPCPs enter the soil, PPCPs can enter the plant through the plant root system [14,15,16]. PPCPs were detected in environmental media as well as in some plants and animals [17,18,19,20,21], such as blocker drugs (diphenhydramine, chlorpheniramine malate, etc.), lipid regulators (atorvastatin calcium, mevastatin, lovastatin, etc.), antiepileptic drugs (carbamazepine, etc.), stimulants (cortisone, testosterone propionate, etc.), blood pressure regulators (nitrendipine, nimodipine, etc.), hypoglycemic drugs (glimepiride, gliclazide, etc.), antibiotics (flumequine, sparfloxacin, etc.), and weight loss drugs (sibutramine, etc.). However, these drugs are widely used in the treatment of human diseases and often produce hazards such as genotoxicity and impairment of physiological functions after long-term exposure. For example, carbamazepine belongs to the anticonvulsant class of drugs, mainly treating seizures and trigeminal neuralgia symptoms, but overdose or long-term use can cause a variety of cardiovascular, neurological, respiratory and urinary symptoms [22]. Sibutramine is an orally administered drug for the treatment of obesity [23], and the use of illegal drugs containing sibutramine can cause cardiovascular toxicity problems [24]. Now, only some of the PPCPs have been shown to accumulate in plants, and most of them have not been shown to be absorbed by plants. A few scholars are conducting studies on the uptake of some drugs by plants, most of them using high concentration levels of PPCPs, and the drug species of PPCPs studied and the experimental subjects were relatively single [25,26,27]; it is difficult to study PPCPs in plants using the same experimental conditions as the environment, and many experimental factors are difficult to control. Therefore, in this study, twenty-seven species of PPCPs were selected from seven major drug species as research objects, and different kinds of sprouts were cultivated in a constant temperature and humidity culture environment to ensure better growth and development of sprouts, which can better study the development pattern of drugs in plants. Sprouts are edible “sprouts” grown from the seeds of various kinds of cereals, legumes, and trees, also known as “living vegetables”. They have a short production cycle, 7–15 days to market, simple technology, a wide range of temperature adaptability, and no fertilization to ensure sufficient moisture. Therefore, the choice of sprouts as experimental subjects allows for better control of experimental conditions and eliminates the need to provide nutrition, thus reducing the influence of other external factors on PPCPs and allowing for better exploration of drug uptake and transport by plants. Moreover, this experiment was designed to study the uptake capacity, translocation, and accumulation degree of drugs in plants grown in different concentrations of PPCPs medium, to clarify which drugs have strong uptake and accumulation capacity in plants, and to provide some guidance for the management and disposal of environmental pollutants, as well as for human food safety.

## 2. Methods and Materials

### 2.1. Reagents

PPCPs mixed standard solution contains 27 drugs, including nisoldipine (Nis), minoxidil (Min), nimodipine (Nim), nicardipine hydrochloride (Nic), indapamide (Ind), lovastatin (Lov), mevastatin (Mev), amantadine (Ama), diphenhydramine (Dip), chlorpheniramine maleate (Chl), carbamazepine (Car), sibutramine (Sib), haemosibutramine (Hom-sib), chlorosibutramine (Chl-sib), N-monodesmethyl sibutramine (N-sib), N, N-bis-desmethyl sibutramine (N, N-sib), glibenclamide (Glibe), glimepiride (Glime), gliclazide (Glie), regeneronide (Rep), danazol (Dan), testosterone propionate (Oreton), cortisone (Cor), flumequine (Flu), sparfloxacin (Spa), atorvastatin calcium (Ato), nicorandipine (Nit). All of them were purchased from Beijing Manhag Biotechnology Co. A mixed standard stock solution of 1 mg/mL was prepared in methanol and placed at −20 °C for standby. Acetonitrile (HPLC grade) was purchased from Fisher, USA. Methanol (HPLC grade) was purchased from Anpel, Shanghai, China. Sodium chloride (analytical purity) was purchased from Tianjin Yongda Chemical Reagent Co. The deionized water was prepared by a Milli-Q ultrapure water preparation instrument (Millipore Corporation, Burlington, MA, USA).

### 2.2. Apparatus

LCMS-8050-Liquid Chromatography Mass Spectrometer (SHIMADZU, Kyoto, Japan), AdVantageXL-70 Vacuum Freeze Dryer (SP SCICENTIFIC, Warminster, PA, USA), Milli-Q Ultrapure Water Preparation Instrument (Millipore, Burlington, MA, USA), HYM-1500-S constant temperature and humidity box (Shanghai Huyueming Scientific Instrument Co., Ltd., Shanghai, China).

### 2.3. Plant Cultivation and Treatment

A batch of sprouting seeds of radish, buckwheat, and okra was purchased from a plant dealer to remove dry stirred, deformed, and damaged seeds. Each group weighed 50.0 g and washed the seeds with deionized water 3times. Then, they were soaked in deionized water of 2–3 times the volume of the seeds for 24 h, drained off the excess water, spread the seeds evenly in the seedling tray, covered with seedling paper, and sprayed with deionized water 2–3 times a day until the seeds germinated. When the buds grow to about 1 cm, remove the nursery paper and add 500 mL of culture solution to the culture tray. Each plant was exposed to three concentration levels of PPCPs at 0.5, 5.0, and 50.0 ng/mL. The control group was the plant culture group without PPCPs drug addition. Throughout the incubation process, all trays were placed in a constant temperature and humidity plant incubator. Cultures were incubated in relative air humidity between 65.0% and 75.0%.; 25 °C, 14 h of light, and 14 °C, 10 h of darkness for 12 days alternately. To avoid the excessive consumption of PPCPs, their degradation, and algal blooms, the culture solution was changed every 3 days in all experimental groups. After 12 days of growth, the plants were removed from the culture trays and washed 2–3 times with deionized water to remove surface residues. Then, they were divided into three parts: roots, stems, and leaves, freeze-dried for 48 h, ground into a powder with a coffee grinder, and stored at −20 °C for backup.

### 2.4. Determination of PPCPs in Plants

#### 2.4.1. Pre-Treatment of Plant Samples

We weighed 0.2 g of lyophilized plant sample powder in a 50 mL centrifuge tube and added 1.8 mL of deionized water to restore the homogenized state of the plant sample; we then added 5.0 g NaCl, 20 mL acetonitrile, vortexed and mixed well. It was then ultrasonicated and extracted for 20 min, centrifuged at 10,000 rpm for 5 min. The extract was taken at 5 mL, nitrogen blown until nearly dry, and the residue was re-dissolved with 1 mL of methanol, then 9 mL of ultrapure water was added and mixed, passed through an HLB solid phase extraction column (150 mg, waters, Milford, MA, USA) pre-treated with 7 mL of methanol and 7 mL of deionized water; the extract was eluted with 7 mL of methanol, then the methanol extract was placed in a water bath at 40 °C, nitrogen blown until nearly dry, and the residue was passed through 1 mL. The residue was re-dissolved with 1 mL of 0.1% formic acid water-acetonitrile solution (1:1, *v*/*v*), passed through 0.22 μm nylon filter membrane, and put on the machine for measurement.

#### 2.4.2. Instrument Methods

In this study, PPCPs in plant tissues were identified and quantified by high-performance liquid chromatography-tandem mass spectrometry. The analysis was performed using UPLC-MS/MS system, including a CBM-20A system controller (Shimadzu, Kyoto, Japan), binary LC-30AD transfer pump, DGU-20A5R vacuum degasser, CTO-30A column furnace, SIL-30AC automatic sampler, and a triple quadrupole mass spectrometer detector Shimadzu LC MS-8050 coupled to the LC System. UPLC18@ column (2.1 × 100 mm, 1.7 µm; Waters Co., Wexford, Ireland) was used for chromatographic separation with column temperature at 40 °C. The mobile phase B was acetonitrile, and the mobile phase A was 0.1% formic acid water at a flow rate of 0.4 mL/min. The mobile phase gradient started at 10.0% organic phase B and was maintained for 0.5 min, ramped up to 90.0% organic phase B at 6 min and was maintained for 2 min, and organic phase B returned to 10.0% in 0.1 min and was maintained for 2 min until the end. The autosampler temperature was maintained at 4 °C and the injection volume was 5 μL.

#### 2.4.3. Quantitative Analysis

A series of matrix-matched mixed standard solutions were prepared, and the standard curve was established with the quantile peak area as the vertical coordinate (*y*) and the corresponding mass concentration as the horizontal coordinate (*x*). A low-level addition was taken in the blank matrix, the method limit of detection (LOD) was set at S/N > 3, and the method limit of quantification (LOQ) was set at S/N > 10. In this experiment, three spiked levels of 2, 20, and 80 ng/mL were used for each compound in six replicate determination experiments to determine the average recovery and precision of each drug.

Excel software was used to analyze the data. Results are expressed as mean ± SD (n = 3). 

## 3. Results and Discussion

### 3.1. Linearity Equation, Detection Limit, and Quantification Limit

As shown in Table 1, in this experiment, the linear correlation coefficients (R^2^) for all drugs were greater than 0.99. Three spiked levels of 2, 20, and 80 ng/mL were performed for each compound with six replicate measurement experiments. All drug recoveries ranged from 81.1% (flumequine) to 122.3% (N-desmethyl sibutramine), and reproducibility ranged from 5.3% (carbamazepine) to 11.6% (diphenhydramine). The detection limits of the drugs ranged from 0.4 (repaglinide) to 6.2 ng/g (flumequine) (dry weight), and the quantification limits ranged from 1.2 (chlorpheniramine maleate) to 11.6 ng/g (gliclazide) (dry weight).

### 3.2. Evaluation of Plant Growth Status

**Figure 2:**
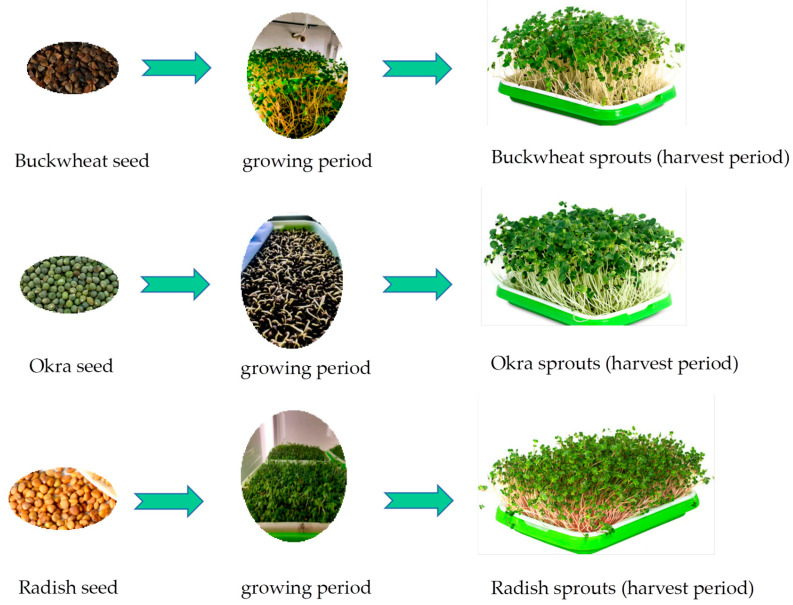
Growth of radish, buckwheat and okra sprouts in 50 ng/mL PPCPs medium.

By observing the growth and development of radish, buckwheat, and okra in the blank group and the three experimental groups for 12 days, the vegetables in the three experimental groups showed no significant difference in growth number, growth height and the state of the plants themselves compared with the sprouts in the blank group, and there was no yellowing of the leaves or death of the vegetables. The addition of PPCPs showed no phytotoxic or other effects on the vegetables, as shown in Figure 2.

### 3.3. Uptake of Different Concentrations of PPCPs by Plants

The uptake of PPCPs in the tissues of three types of vegetables at different culture concentrations was analyzed after 12 days of incubation. Three vegetables were grown in cultures containing 0.5 ng/mL PPCPs; most of the PPCPs were detected in the plants. Among 27 PPCPs, 11 PPCPs were found in radish, 16 PPCPs in buckwheat, and 16 PPCPs in okra as shown in Table 2. The results showed that some PPCPs were absorbed and accumulated by plants under low concentration PPCPs culture. PPCPs absorbed include nicardipine hydrochloride, amantadine, diphenhydramine, chlorpheniramine maleate, carbamazepine, sibutramine, haemosibutramine, chlorosibutramine, N-monodesmethyl sibutramine, N, N-desmethyl sibutramine, gliphenylurea, glimepiride, repaglinide, flumequine, sparfloxacin, and atorvastatin calcium. Among vegetables in cultures containing 5.0 ng/mL PPCPs, 13 PPCPs in radish sprouts, 16 PPCPs in buckwheat sprouts, and 18 PPCPs in okra sprouts. Among sprouts in cultures containing 50.0 ng/mL PPCPs, 19 PPCPs were found in radish, 22 PPCPs in buckwheat, and 20 PPCPs in okra. It showed that as the content of PPCPs in the culture solution increased, the amount of PPCPs absorbed by the plants also showed an increasing trend.

**Figure 3:**
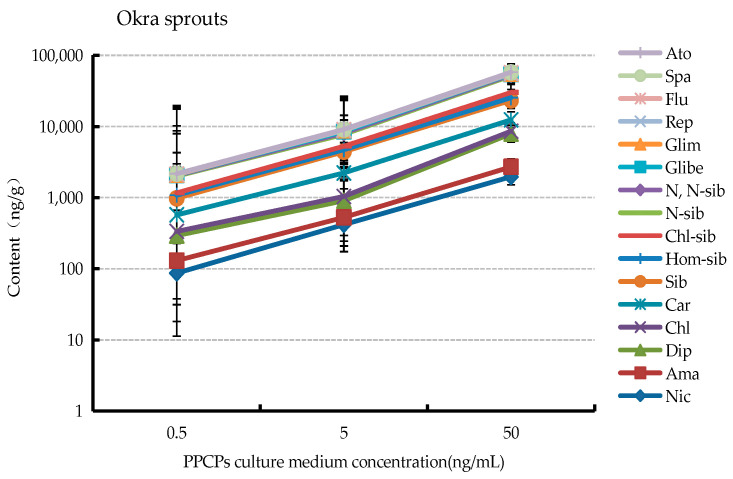
Changes in drug absorption and accumulation of three kinds of Okra root in PPCPs culture medium at 0.5, 5.0 and 50.0 ng/mL.

As shown in Figure 3, the analysis of PPCPs in sprout roots under 0.5, 5.0, and 50.0 ng/mL PPCPs cultures revealed that the accumulation of drug uptake in plant roots showed a significant increase with the increase in PPCPs concentration in the culture solution. In addition, a similar phenomenon was observed in the stems and leaves of sprouts. It showed that the concentration of external PPCPs had a significant effect on the uptake and accumulation of plant drugs.

### 3.4. Accumulation and Distribution of PPCPs in Plants

**Figure 4:**
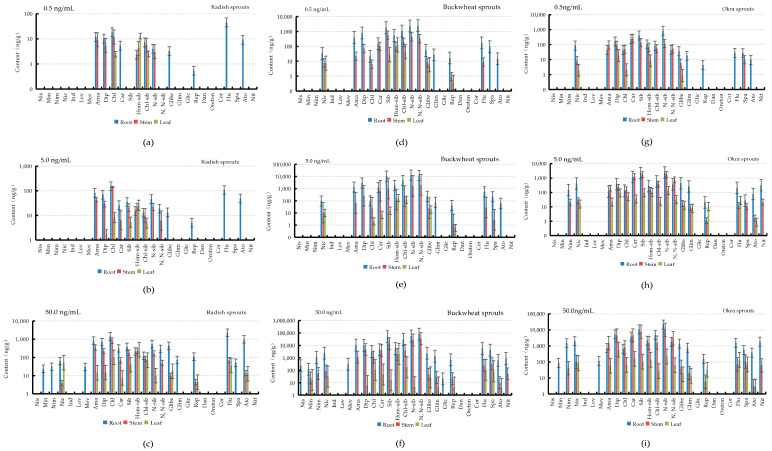
Distribution of content in roots, stems and leaves of radish (**a**–**c**), buckwheat (**d**–**f**) and okra (**g**–**i**) cultured at three concentration levels of PPCPs: low (0.5 ng/mL), medium (5.0 ng/mL) and high (50 ng/mL).

To understand the distribution and accumulation of PPCPs drugs in plants, the mature vegetables were washed in deionized water and separated by root, stem, and leaf, and the PPCPs in each tissue of vegetables were analyzed and determined by LC-MS/MS, respectively. In 50.0 ng/mL PPCPs culture solution, statistical analysis revealed that the content of PPCPs in radish roots ranged from 23.8 (minoxidil) to 2.3 × 10^3^ ng/g (flumequine), the content ranged from 3.9 (nicardipine hydrochloride) to 8.4 × 10^2^ ng/g (chlorpheniramine maleate) in the stem, and the content ranged from 4.4 (repaglinide) to 2.7 × 10^2^ ng/g (haemosibutramine) in the leaf. The distribution of amantadine, diphenhydramine, chlorpheniramine maleate, carbamazepine, chlorosibutramine, monodesmethyl sibutramine, glibenclamide, repaglinide, flumequine, and atorvastatin calcium was root > stem > leaf in radish. The distribution of haemonosibutramine was leaf > stem > root in radish. The distribution of nicardipine hydrochloride and repaglinide were root > leaf > stem, while minoxidil, nimodipine, sparfloxacin and glimepiride were only distributed in the root. Nisoldipine, cindapamide, lovastatin, gliclazide, danazol, testosterone propionate, cortisone, and nicorandipine were not distributed in the root, stem, or leaves, as shown in Figure 4c. The content of PPCPs in the roots of buckwheat ranged from 20.6 (gliclazide) to 8.1 × 10^4^ ng/g (N, N-desmethyl sibutramine). The content of PPCPs in stems ranged from 10.0 (atorvastatin calcium) to 3.5 × 10^4^ ng/g (N, N-bisdemethylsibutramine). The content of PPCPs in leaves ranged from 3.1 (atorvastatin calcium) to 2.0 × 10^3^ ng/g (haumosibutramine). Among them, minoxidil, nicardipine hydrochloride, diphenhydramine, chlorpheniramine maleate, carbamazepine, sibutramine, chlorosibutramine, N-desmethyl sibutramine, gliphenylurea, glimepiride, repaglinide, flumequine, sparfloxacin, and atorvastatin calcium were distributed as root > stem > leaf. The distribution of haemonosibu tramine was root > leaf > stem. In contrast, nisoldipine, mevastatin, and gliclazide were distributed only in the roots. Nimodipine, N, N-bisdemethylsibutramine, and nitrendipine were only distributed in roots and stems. Epidapamide, lovastatin, danazol, testosterone propionate, and cortisone were not distributed in roots, stems, and leaves, as shown in Figure 4f. PPCPs content in the roots of okra ranged from 84.8 (minoxidil) to 2.1 × 10^4^ ng/g (N-desmethyl sibutramine). The content of PPCPs in stems ranged from 3.2 (atorvastatin calcium) to 1.0 × 10^4^ ng/g (N-desmethyl sibutramine). The content of PPCPs in leaves ranged from 2.7 (atorvastatin calcium) to 5.9 × 10^2^ (diphenhydramine) ng/g. Among them, nicardipine hydrochloride, diphenhydramine, sibutramine, haumosibutramine, chlorosibutramine, N-monodesmethyl sibutramine, glibenclamide, glimepiride, sparfloxacin, and atorvastatin calcium distribution in buckwheat as root > stem > leaf. Amantadine, chlorpheniramine maleate, carbamazepine, and N, N-desmethyl sibutramine were distributed as stem > root > leaf. Flumequine distribution was root > leaf > stem. Nimodipine and nitrendipine were only distributed in the root and stem. Minoxidil, as well as mevastatin, were only distributed in the roots, while indapamide, lovastatin, gliclazide, danazol, testosterone propionate, and cortisone were not distributed in the roots, stems, and leaves as shown in Figure 4i, and the distribution of PPCPs absorbed by three vegetables was similar in roots, stems, and leaves under 0.5 and 5.0 ng/mL PPCPs culture conditions, as shown in Figure 4a,b,d,e,g,h. Although there was no obvious pattern in the distribution of different PPCPs within plant tissues, the analysis of the PPCPs in different tissues of different plants demonstrated that there was a clear uptake transfer of PPCPs by plants. Moreover, the trend of absorption and transfer would become more and more obvious with the increase in external environmental pollutant concentration. Under 50 ng/mL PPCPs incubation conditions, some of PPCPs were detected only in the roots of the plants, and even some of PPCPs were not absorbed; it may be that some of the PPCPs did not reach the concentration for plant uptake or the contact time between the plants and the PPCPs was not sufficient for plant uptake. The phenomenon that plants differ in the extent of uptake and accumulation of different PPCPs is consistent with the results of Zhang’s study [28]. Biological properties such as lipid content and carbohydrate content of the root system, physicochemical properties of PPCPs (K_ow_, molecular size, etc.) and environmental conditions of the medium may affect the uptake of PPCPs by plants. The lipid and carbohydrate contents of the root cell wall and the permeability of the root cell membrane have been reported in several related studies as the main biological factors for the uptake of PPCPs by plants into the root system [29,30]. Moreover, the physicochemical properties of PPCPs (K_ow_, hydrophobic properties, etc.), and ionic properties (anions, cations, etc.) also significantly influenced the uptake of PPCPs by plants from outside [31]. These factors may influence factors for the phenomenon of different levels of plant uptake and accumulation of different PPCPs in the present study. However, stems, leaves, and even roots are the edible parts of sprouts. In this study, it was found that PPCPs have a significant migration distribution in sprouts, and if sprouts are grown with water contaminated by PPCPs, they are likely to accumulate in the plant and enter the human body, posing a potential threat to human health.

### 3.5. Bioconcentration Factor (BCF) Evaluation of PPCPs in Plant Tissues

Bioconcentration factors are important indicators for evaluating phytoremediation, uptake, and translocation, and to describe the accumulation of compounds in the organism [27]. The bioconcentration factor was calculated as the ratio of the concentration of the compound in the dry weight plant tissue to the concentration of the compound added to the culture medium (1) [32].

(1) $$\mathrm{BCF}\left(L/kg\right)=\frac{Concentration\;of\;compounds\;in\;plant\;tissues\left(\mu g/kg\right)}{Concentration\;of\;compounds\;in\;the\;culture\;medium\left(\mu g/L\right)}$$

**Figure 5:**
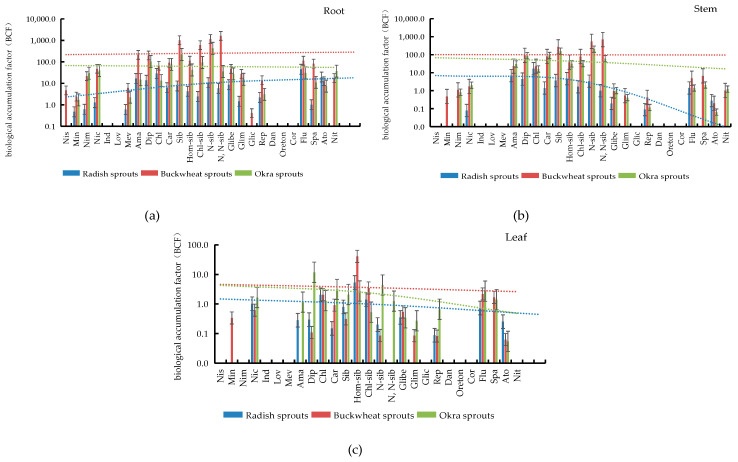
Differences of PPCPs bioenrichment in roots (**a**), stems (**b**) and leaves (**c**) of three vegetables.

As shown in Table 3, the analysis of three vegetables under 50.0 ng/mL PPCPs culture conditions revealed that all PPCPs absorbed by the vegetables bioaccumulated significantly in the roots of the plants. For example, the BCF of amantadine in the roots of sprouts ranged from 14.9 to 2.1 × 10^2^ L/kg, the BCF of diphenhydramine ranged from 13.9 to 2.0 × 10^2^ L/kg, and the BCF of chlorpheniramine maleate ranged from 13.4 to 65.6 L/kg, etc. In addition, high bioaccumulation was also observed in the stems and leaves of sprouts, for example, BCF of amantadine in stems ranged from 6.9 to 30.3 L/kg, BCF of diphenhydramine in stems ranged from 4.4 to 92.0 L/kg, and BCF of chlorpheniramine maleate in stems ranged from 16.1 to 22.9 L/kg, etc. Moreover, some differences were found in the bioconcentration factors of most PPCPs in the three vegetables tissues, as shown in Figure 5. The accumulation of PPCPs in buckwheat sprouts and okra sprouts was significantly higher than that in radish sprouts. This is similar to the phenomenon reported by Wu Xiaoqin et al. in which the BCF of PPCPs in pepper leaves was significantly higher than those in lettuce, spinach, and cucumber leaves [32]. It indicates that the absorption, migration, and accumulation of drugs by plants may be related to the species of plants. The translocation and accumulation ability of PPCPs among plant tissues may be related to the difference in lipid content and metabolic detoxification ability of plants [31]. Moreover, different PPCPs also have obvious differences in the same plant tissues. For example, the bioconcentration factors of amantadine, phenazopyridine, chlorpheniramine maleate, sibutramine, haemosibutramine, chlorosibutramine, N-monodesmethyl sibutramine, and N, N-desmethyl sibutramine in buckwheat tissues were significantly higher than those of other PPCPs absorbed by plants. This is similar to the study reported by Herklotz et al. In cabbage grown under hydroponic conditions, the accumulation of sulfamethoxazole was higher than that of carbamazepine and trimethoprim [33]. It indicated that the absorption, translocation, and accumulation of PPCPs by plants may be related to the properties of PPCPs [25,32,34]. In the study of organic pollutant accumulation in vegetation by Simonich et al., it was similarly shown that plant uptake of organic pollutants was influenced by the chemical and physical properties of the pollutants, environmental conditions, and plant species [35].

### 3.6. Translocation of PPCPs in Plant Tissues

After the compound was absorbed by plant roots, the plant may distribute the compound to individual tissues [36]. In this study, translocation factors (TF) were calculated for PPCPs distributed in all plant roots, stems, and/or leaves by analysis of sprouts in the 0.5, 5.0, and 50.0 ng/mL PPCPs groups. TF = C_leaf/stem_/C_root._

**Figure 6:**
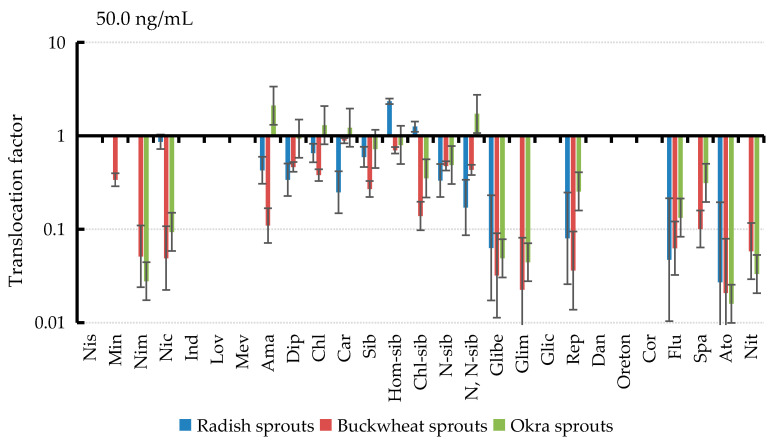
Translocation of PPCPs absorbed by three vegetables grown in medium containing 50.0 ng/mL.

As shown in Figure 6, the translocation factors (TF > 1.0) for amantadine, chlorpheniramine maleate, carbamazepine, and N, N-bisdemethyl sibutramine in the 50.0 ng/mL PPCPs culture group of okra. It showed that these four PPCPs were preferentially transferred to stems or leaves after being absorbed by okra roots. Translocation factors were (TF > 1.0) for haumosibutramine and chlorosibutramine in radish. It showed that it was preferentially transferred to the stem or leaf after being absorbed by the radish roots. Among them, the carbamazepine translocation in okra was consistent with the previously reported carbamazepine translocation in cucumber and ryegrass [37]. The translocation factors of other PPCPs in plants (TF < 1.0) showed that other PPCPs accumulated preferentially in the roots of plants. Furthermore, similar phenomena were observed under 0.5 and 5.0 ng/mL PPCPs culture conditions, as shown in Table 4. It was shown that most of PPCPs accumulated preferentially in the roots after roots uptake and then were transferred to other plant tissues. Moreover, amantadine, chlorpheniramine maleate, carbamazepine, N, N-bisdemethylsibutramine, haemosibutramine and chlorosibutramine were more active in translocation processes in some plants. Partial PPCPs as well as transfer to other plant tissues and organs have also been reported in some drug transport studies. Martínez-Piernas et al. found 17 PPCPs in the leaves of field-grown tomatoes irrigated with reclaimed water at concentrations ranging from 0.04–32 ng/g [38]. Similarly, Zhao et al. showed that long-term application of fertilizer under tillage conditions introduced antibiotics into peanut plants [39]. In the migration of antibiotics in peanut plants, sulfamethoxazole had the highest TF value, followed by enrofloxacin, erythromycin and crotetracycline, while ciprofloxacin had the lowest TF value. Furthermore, in some studies on carbamazepine in plants, different TF values were found for carbamazepine in cabbage, lettuce, and zucchini; and different TF values were found for caffeine in potato and zucchini plants, which may indicate that the translocation of these compounds depends on the plant type [40]. All of these findings were similar to the present study. It was demonstrated that plant migration for PPCPs and migration differences were closely related to the biological properties of the plants themselves, and it could be clearly shown that PPCPs can migrate and accumulate in plants and continuously contaminate tissues and organs in other parts of the plants.

## 4. Conclusions

The results of this study clearly showed that plants exposed to water containing PPCPs were able to take up a variety of PPCPs through the root system and revealed significant differences in the uptake, transport, and accumulation of PPCPs in buckwheat, radish, and okra, a phenomenon that may be related to the molecular mass of PPCPs, hydrophobicity, root lipid content, dissolved organic carbon, pH, and environmental organic matter content. This phenomenon may be related to the molecular mass of PPCPs, hydrophobicity, root lipid content, dissolved organic carbon, pH and environmental organic matter content. However, many current investigations have shown that toxic chemicals not only affect plants, but also endanger human health. The results of this study will not only improve our understanding of PPCPs as a novel contaminant, but also reveal that some therapeutic drugs, such as antibiotics, that protect public health, cannot be completely banned because they are necessary for human and animal health, agriculture, and livestock production. Meanwhile, among the 27 PPCPs used in this study, amantadine, phenazopyridine, chlorpheniramine maleate, sibutramine, haemosibutramine, chlorosibutramine, N-monomethyl sibutramine, N, N-desmethyl sibutramine and carbamazepine bioaccumulated significantly more than other PPCPs in plants. These drugs should be the focus of subsequent studies. It has also been reported in the literature [41] that conventional drinking water and wastewater treatment plants do not have treatment processes specifically for PPCPs and that the existing processes do not completely remove PPCPs. Therefore, monitoring these organic chemicals is important for public health and improving the biological environment, and the presence and trends of these contaminants in water, soil, and plants have been the subject of many environmental studies around the world. We believe that more studies should be conducted to determine the precise transfer pathways and their temporal patterns, and that a better understanding of these factors would help to improve the control of cumulative toxic effects on plants and to reduce the harmful effects of contaminants.

## Figures and Tables

**Table 1:** Validation data of 27 PPCPs in sprouts.

Drugs	Linear Equations	Linear Range (ng/mL)	R^2^	LOD/(ng/g)	LOQ/(ng/g)	Average Recovery Rate (%) (n = 6)		
						2 ng/mL	20 ng/mL	80 ng/mL
nisoldipine	*y* = 108034*x* − 15127.8	0.5–200	0.9997	1.8	5.5	93.6	101.3	96.1
minoxidil	*y* = 473274*x* + 216871	0.1–150	0.9997	1.0	2.8	115.3	97.0	90.8
nimodipine	*y* = 37211.8*x* + 47937.7	1.0–200	0.9999	2.4	7.2	86.0	84.5	90.7
nicardipine hydrochloride	*y* = 109561*x* + 186323	0.1–200	0.9940	1.8	5.5	91.8	103.6	99.6
indapamide	*y* = 71840.3*x* − 15690.5	0.5–200	0.9978	2.0	6.2	86.6	94.1	98.5
lovastatin	*y* = 13679.3*x* + 3255.78	1.0–100	0.9970	2.8	8.4	95.7	92.1	91.1
mevastatin	*y* = 27393.2*x* − 14427.6	0.5–100	0.9980	2.5	7.4	106.3	84.6	90.3
amantadine	*y* = 120767*x* + 418807	0.1–200	0.9983	1.8	5.5	101.2	85.3	98.4
diphenhydramine	*y* = 207602*x* − 1168800	1.0–300	0.9960	1.5	4.6	101.0	90.8	88.6
chlorpheniramine maleate	*y* = 521211*x* + 473248	0.1–200	0.9971	0.9	2.8	94.8	90.8	105.1
carbamazepine	*y* = 5579163*x* + 750344	0.1–100	0.9963	0.1	0.3	96.5	90.5	86.3
sibutramine	*y* = 29292.6*x* + 1717.77	1.0–200	0.9980	2.5	7.4	120.4	94.9	110.1
haemosibutramine	*y* = 778095*x* + 456360	0.5–150	0.9984	0.6	1.8	103.8	91.6	95.6
chlorosibutramine	*y* = 173778*x* − 500933	1.0–100	0.9962	1.8	5.6	94.8	90.8	93.4
N-monodesmethyl sibutramine	*y* = 8757.68*x* + 7416.21	0.5–100	0.9981	3.1	9.8	122.3	103.7	96.7
N, N-bis-desmethyl sibutramine	*y* = 418080*x* + 110723	0.5–100	0.9997	1.0	3.0	81.7	96.2	96.3
glibenclamide	*y* = 67746.8*x* + 3575.80	1.0–150	0.9963	2.0	6.2	97.6	93.5	102.3
glimepiride	*y* = 29710.4*x* + 2040.04	1.0–150	0.9912	2.5	7.3	107.1	84.8	111.2
gliclazide	*y* = 290295*x* + 4531.97	1.0–100	0.9999	1.5	4.6	97.7	100.6	89.6
repaglinide	*y* = 533270*x* + 8743.73	0.1–100	0.9973	0.9	2.7	80.8	86.9	90.6
danazo	*y* = 681958*x* + 14331.9	0.1–100	0.9964	0.8	2.5	93.6	106.2	93.8
testosterone propionate	*y* = 28410.2*x* − 4312.44	0.5–100	0.9983	2.5	7.4	110.0	113.8	108.3
cortisone	*y* = 235080*x* + 10535.7	0.5–100	0.9961	1.5	4.6	103.9	84.8	90.8
flumequine	*y* = 23557*x* − 557.311	1.0–100	0.9983	2.7	8.1	86.9	81.1	95.3
sparfloxacin	*y* = 79048.7*x* − 40180.1	0.5–200	0.9982	2.0	6.2	86.4	84.5	88.6
atorvastatin calcium	*y* = 217752*x −* 455238	1.0–200	0.9970	1.5	4.6	95.3	99.6	94.6
nicorandipine	*y* = 14159.6*x* + 11150.2	1.0–200	0.9998	2.8	8.4	103.3	94.3	90.5

**Table 2:** Dry weight content of drugs in the roots of three vegetables cultured in 0.5, 5 and 50 ng/mL PPCPs medium (n = 3).

Drug	Radish Root/(ng/g)			Buckwheat Root/(ng/g)			Okra Root/(ng/g)		
	0.5 ng/mL	5 ng/mL	50 ng/mL	0.5 ng/mL	5 ng/mL	50 ng/mL	0.5 ng/mL	5 ng/mL	50 ng/mL
Ato	9.1 ± 0.5	48.6 ± 2.0	9.8 × 102 ± 54.9	14.7 ± 0.9	63.8 ± 5.0	6.3 × 102 ± 34.7	9.5 ± 0.4	77.4 ± 1.7	3.7 × 102 ± 19.6
Flu	45.3 ± 1.5	106.7 ± 7.3	2.3 × 103 ± 163.3	1.7 × 102 ± 11.7	5.5 × 102 ± 29.2	5.7 × 103 ± 273.6	28.2 ± 0.7	2.1 × 102 ± 8.8	1.5 × 103 ± 48.0
Rep	4.9 ± 0.2	15.1 ± 0.5	1.1 × 102 ± 5.9	16.6 ± 0.8	42.5 ± 3.3	7.0 × 102 ± 30.8	4.3 ± 0.2	20.2 ± 0.6	1.5 × 102 ± 2.4
Ama	12 ± 0.9	82.1 ± 4.6	8.4 × 102 ± 33.6	4.0 × 102 ± 20.4	1.4 × 102 ± 10.4	1.1 × 104 ± 231.0	43.5 ± 1.0	1.1 × 102 ± 6.5	7.5 × 102 ± 48
Dip	10.2 ± 0.4	69.2 ± 3.5	7.0 × 102 ± 30.8	8.0 × 102 ± 33.6	2.9 × 103 ± 78.3	9.9 × 103 ± 336.6	1.6 × 102 ± 4.2	3.8 × 102 ± 19.8	5.2 × 103 ± 208.0
Chl	18.3 ± 0.3	1.5 × 102 ± 1.5	1.4 × 103 ± 75.6	21.3 ± 1.02	99.6 ± 6.5	3.3 × 103 ± 85.8	42.6 ± 1.7	1.3 × 102 ± 3.5	6.7 × 102 ± 36.2
Car	5.4 ± 0.5	26.3 ± 0.9	3.1 × 102 ± 22.3	1.5 × 102 ± 9.2	1.2 × 103 ± 69.6	4.5 × 103 ± 148.5	2.4 × 102 ± 7.2	1.2 × 103 ± 69.6	3.8 × 103 ± 68.4
Hom-sib	2.3 ± 0.1	15.2 ± 0.4	2.1 × 102 ± 4.6	5.3 × 102 ± 20.1	1.8 × 103 ± 66.6	5.6 × 103 ± 140.0	1.0 × 102 ± 2.3	2.8 × 102 ± 13.4	2.1 × 103 ± 157.5
Chl-sib	7.2 ± 0.7	12.8 ± 1.1	1.2 × 102 ± 11.9	1.1 × 103 ± 40.7	4.7 × 103 ± 197.4	3.0 × 104 ± 2430.0	84 ± 5.8	5.8 × 102 ± 129.6	4.7 × 103 ± 103.4
Glibe	3.2 ± 0.3	13.2 ± 0.6	4.3 × 102 ± 13.8	53.2 ± 3.2	236.3 ± 9.2	2.3 × 103 ± 131.1	35.9 ± 2.4	4.4 × 102 ± 18.9	1.4 × 103 ± 39.2
N-sib	4.0 ± 0.3	46.3 ± 4.4	5.4 × 102 ± 27.5	2.3 × 103 ± 112.7	1.3 × 104 ± 663.0	5.8 × 104 ± 3596	8.4 × 102 ± 62.2	2.5 × 103 ± 135.0	2.1 × 104 ± 1848
N, N-sib	/	18.7 ± 0.8	2.9 × 102 ± 22.3	2.5 × 103 ± 67.5	1.2 × 104 ± 744.0	8.1 × 104 ± 2673	44.4 ± 1.3	3.6 × 102 ± 7.56	1.8 × 103 ± 48.6
Sib	/	34.8 ± 1.5	3.8 × 102 ± 25.8	1.8 × 103 ± 55.8	1.1 × 104 ± 451.0	5.1 × 104 ± 2856	4.1 × 102 ± 33.6	2.2 × 103 ± 156.2	1.1 × 104 ± 484
Glim	/	/	73.0 ± 5.4	26.8 ± 2.1	76.2 ± 6.2	1.4 × 103 ± 96.6	17.9 ± 1.2	2.6 × 102 ± 15.3	7.7 × 102 ± 60.8
Spa	/	/	50.7 ± 2.1	93.9 ± 5.1	2.2 × 102 ± 11.4	4.1 × 103 ± 241.9	26.8 ± 1.6	27.1 ± 2.4	5.5 × 102 ± 37.4
Nic	/	/	63.6 ± 4.1	32.9 ± 1.7	95.0 ± 2.7	2.4 × 103 ± 172.8	86.7 ± 8.8	4.2 × 102 ± 22.3	2.0 × 103 ± 154.0
Nim	/	/	30.8 ± 1.0	/	/	1.1 × 103 ± 28.6	/	1.5 × 102 ± 10.2	1.5 × 103 ± 112.5
Min	/	/	23.8 ± 0.9	/	/	1.2 × 102 ± 10.0	/	/	84.8 ± 6.2
Mev	/	/	30.2 ± 2.5	/	/	3.1 × 102 ± 14.3	/	/	1.1 × 102 ± 7.15
Nit	/	/	/	/	/	8.8 × 102 ± 76.6	/	3.2 × 102 ± 17.9	1.8 × 103 ± 198.0
Nis	/	/	/	/	/	2.3 × 102 ± 11.0	/	/	/
Glic	/	/	/	/	/	20.6 ± 0.6	/	/	/
Dan	/	/	/	/	/	/	/	/	/
Oreton	/	/	/	/	/	/	/	/	/
Cor	/	/	/	/	/	/	/	/	/
Ind	/	/	/	/	/	/	/	/	/
Lov	/	/	/	/	/	/	/	/	/

“/” not detected.

**Table 3:** Biological concentration factors in roots, stems and leaves of three kinds of vegetables in 50 ng/mL PPCPs medium (n = 3).

Drug	Radish Sprouts			Buckwheat Sprouts			Okra Sprouts		
	root/(L/kg)	stem/(L/kg)	leaf/(L/kg)	root/(L/kg)	stem/(L/kg)	leaf/(L/kg)	root/(L/kg)	stem/(L/kg)	leaf/(L/kg)
Ato	19.66 ± 1.10	0.28 ± 0.01	0.25 ± 0.01	12.59 ± 0.69	0.20 ± 0.01	0.06 ± 0.01	7.44 ± 0.39	0.06 ± 0.01	0.05 ± 0.01
Flu	45.37 ± 3.22	1.41 ± 0.10	0.73 ± 0.04	113.13 ± 5.40	4.90 ± 0.43	2.19 ± 0.13	30.93 ± 0.99	1.41 ± 0.08	2.70 ± 0.29
Rep	2.21 ± 0.12	0.09 ± 0.01	0.09 ± 0.01	13.96 ± 0.62	0.42 ± 0.04	0.08 ± 0.01	3.06 ± 0.05	0.12 ± 0.01	0.66 ± 0.08
Ama	16.87 ± 0.68	6.92 ± 0.43	0.28 ± 0.02	210.94 ± 4.40	23.08 ± 1.74	/	14.92 ± 0.95	30.29 ± 2.29	1.15 ± 0.11
Dip	13.93 ± 0.61	4.42 ± 0.44	0.29 ± 0.03	198.13 ± 6.70	91.90 ± 11.13	0.11 ± 0.01	104.06 ± 4.20	89.05 ± 3.53	11.77 ± 1.70
Chl	28.77 ± 1.56	16.77 ± 0.89	2.04 ± 0.09	65.64 ± 1.70	22.88 ± 1.46	2.05 ± 0.13	13.39 ± 0.72	16.11 ± 0.59	1.30 ± 0.11
Car	6.24 ± 0.45	1.40 ± 0.07	0.15 ± 0.01	90.65 ± 2.99	79.62 ± 4.68	0.91 ± 0.06	76.11 ± 1.40	89.99 ± 4.59	3.10 ± 6.23
Hom-sib	4.25 ± 0.09	4.63 ± 0.31	5.32 ± 0.31	112.89 ± 2.80	38.19 ± 3.12	40.66 ± 2.28	42.13 ± 3.20	30.91 ± 1.73	2.74 ± 0.28
Chl-sib	2.44 ± 0.24	1.65 ± 0.13	1.41 ± 0.09	598.82 ± 48.50	79.47 ± 7.15	3.46 ± 0.12	93.07 ± 2.04	32.12 ± 1.46	0.53 ± 0.06
Glibe	8.64 ± 0.28	0.19 ± 0.01	0.35 ± 0.02	46.85 ± 2.67	0.96 ± 0.07	0.54 ± 0.05	27.61 ± 0.78	1.01 ± 0.02	0.34 ± 0.03
N-sib	10.70 ± 0.55	3.36 ± 0.13	0.20 ± 0.01	1154.50 ± 71.58	549.91 ± 25.10	0.09 ± 0.01	426.60 ± 37.54	203.64 ± 14.60	4.36 ± 0.27
N, N-sib	5.84 ± 0.45	1.00 ± 0.10	/	1611.96 ± 53.26	696.99 ± 80.30	/	35.48 ± 0.95	59.70 ± 4.90	1.25 ± 0.17
Sib	7.51 ± 0.51	3.66 ± 0.41	0.80 ± 0.08	1016.98 ± 56.95	273.28 ± 36.07	0.31 ± 0.01	218.84 ± 9.63	156.23 ± 9.70	2.09 ± 0.33
Glim	1.46 ± 0.11	/	/	27.57 ± 1.90	0.53 ± 0.05	0.08 ± 0.01	15.38 ± 1.21	0.41 ± 0.02	0.27 ± 0.03
Spa	1.01 ± 0.04	/	/	82.99 ± 4.89	6.68 ± 0.39	1.67 ± 0.07	11.06 ± 0.75	2.07 ± 0.09	1.40 ± 0.11
Nic	1.27 ± 0.08	0.08 ±	1.02 ± 0.05	47.59 ± 3.43	1.72 ± 0.11	0.62 ± 0.03	39.28 ± 3.02	2.02 ± 0.07	1.65 ± 0.14
Nim	0.62 ± 0.02	/	/	21.60 ± 0.56	1.11 ± 0.06	/	29.53 ± 2.21	0.82 ± 0.04	/
Min	0.48 ± 0.02	/	/	2.40 ± 0.20	0.47 ± 0.03	0.34 ± 0.02	1.70 ± 0.12	/	/
Mev	0.60 ± 0.05	/	/	6.18 ± 0.29	/	/	2.26 ± 0.15	/	/
Nit	/	/	/	17.65 ± 1.54	1.03 ± 0.07	/	36.84 ± 4.05	1.22 ± 0.06	/
Nis	/	/	/	4.70 ± 0.23	/	/	/	/	/
Glic	/	/	/	0.41 ± 0.01	/	/	/	/	/
Dan	/	/	/	/	/	/	/	/	/
Oreton	/	/	/	/	/	/	/	/	/
Cor	/	/	/	/	/	/	/	/	/
Ind	/	/	/	/	/	/	/	/	/
Lov	/	/	/	/	/	/	/	/	/

“/” not detected.

**Table 4:** Translocation factor (TF) values of target PPCPs in vegetables grown in nutrient solution containing 0.5 (a), 5.0 (b), and 50.0 (c) ng/mL.

Drug	0.5 ng/mL			5 ng/mL			50 ng/mL		
	Radish	Buckwheat	Okra	Radish	Buckwheat	Okra	Radish	Buckwheat	Okra
Ama	0.76	0.06	2.15	0.52	0.05	1.98	0.43	0.11	2.11
Dip	0.54	0.09	0.66	0.44	0.09	0.84	0.34	0.46	0.97
Chl	0.78	0.30	1.21	0.98	0.29	1.51	0.65	0.38	1.30
Car	/	0.67	1.09	0.24	0.54	1.07	0.25	0.89	1.22
Sib	/	0.33	0.34	0.75	0.10	0.83	0.59	0.27	0.72
Hom-sib	7.32	0.42	0.72	3.13	0.16	0.86	2.34	0.70	0.80
Chl-sib	1.12	0.21	0.64	1.17	0.08	0.69	1.25	0.14	0.35
N-sib	0.77	0.19	0.14	0.48	0.13	0.77	0.33	0.48	0.49
N, N-sib	/	0.13	1.18	0.33	0.17	2.13	0.17	0.43	1.72
Glibe	/	0.26	0.19	/	0.19	0.07	0.06	0.03	0.05
Nic	/	0.46	0.12	/	0.22	0.10	0.86	0.05	0.09
Flu	/	0.05	/	/	0.05	0.19	0.05	0.06	0.13
Spa	/	/	0.36	/	0.01	0.39	/	0.10	0.31
Rep	/	0.08	/	/	0.04	0.56	0.08	0.04	0.25
Ato	/	/	/	/	/	0.03	0.03	0.02	0.02
Nit	/	/	/	/	/	0.07	/	0.06	0.03
Nim	/	/	/	/	/	0.14	/	0.05	0.03
Glim	/	/	/	/	/	0.07	/	0.02	0.04
Min	/	/	/	/	/	/	/	0.34	/
Glic	/	/	/	/	/	/	/	/	/
Nis	/	/	/	/	/	/	/	/	/
Dan	/	/	/	/	/	/	/	/	/
Oreton	/	/	/	/	/	/	/	/	/
Cor	/	/	/	/	/	/	/	/	/
Ind	/	/	/	/	/	/	/	/	/
Lov	/	/	/	/	/	/	/	/	/
Mev	/	/	/	/	/	/	/	/	/

“/” not migrated.
